# Therapeutic benefit of balneotherapy and hydrotherapy in the management of fibromyalgia syndrome: a qualitative systematic review and meta-analysis of randomized controlled trials

**DOI:** 10.1186/ar4603

**Published:** 2014-07-07

**Authors:** Johannes Naumann, Catharina Sadaghiani

**Affiliations:** 1 Interdisciplinary Center for Treatment and Research in Balneology, Institute for Environmental Health Sciences and Hospital Infection Control, Medical Center University of Freiburg, Breisacher Straße 115b, Freiburg im Breisgau 79106, Germany

## Abstract

**Introduction:**

In the present systematic review and meta-analysis, we assessed the effectiveness of different forms of balneotherapy (BT) and hydrotherapy (HT) in the management of fibromyalgia syndrome (FMS).

**Methods:**

A *s*ystematic literature search was conducted through April 2013 (Medline via Pubmed, Cochrane Central Register of Controlled Trials, EMBASE, and CAMBASE). Standardized mean differences (SMDs) and 95% confidence intervals (CIs) were calculated using a random-effects model.

**Results:**

Meta-analysis showed moderate-to-strong evidence for a small reduction in pain (SMD −0.42; 95% CI [−0.61, −0.24]; *P* < 0.00001; I^2^ = 0%) with regard to HT (8 studies, 462 participants; 3 low-risk studies, 223 participants), and moderate-to-strong evidence for a small improvement in health-related quality of life (HRQOL; 7 studies, 398 participants; 3 low-risk studies, 223 participants) at the end of treatment (SMD −0.40; 95% CI [−0.62, −0.18]; *P* = 0.0004; I^2^ = 15%). No effect was seen at the end of treatment for depressive symptoms and tender point count (TPC).

BT in mineral/thermal water (5 studies, 177 participants; 3 high-risk and 2 unclear risk studies) showed moderate evidence for a medium-to-large size reduction in pain and TPC at the end of treatment: SMD −0.84; 95% CI [−1.36, −0.31]; *P* = 0.002; I^2^ = 63% and SMD −0.83; 95% CI [−1.42, −0.24]; *P* = 0.006; I^2^ = 71%. After sensitivity analysis, and excluding one study, the effect size for pain decreased: SMD −0.58; 95% CI [−0.91, −0.26], *P* = 0.0004; I^2^ = 0. Moderate evidence is given for a medium improvement of HRQOL (SMD −0.78; 95% CI [−1.13, −0.43]; *P* < 0.0001; I^2^ = 0%). A significant effect on depressive symptoms was not found. The improvements for pain could be maintained at follow-up with smaller effects.

**Conclusions:**

High-quality studies with larger sample sizes are needed to confirm the therapeutic benefit of BT and HT, with focus on long-term results and maintenance of the beneficial effects.

## Introduction

Fibromyalgia syndrome (FMS) is a debilitating condition of almost unknown etiology and pathogenesis that is characterized by widespread musculoskeletal pain and tenderness, as well as secondary symptoms like fatigue, depression, irritable bowel syndrome and sleep disturbances. A standard therapy regimen is lacking and the condition causes high direct and indirect costs (for example, health care use, sick leave) [1]. In a survey of the German population using the modified American College of Rheumatology (ACR) 2010 preliminary diagnostic criteria for FMS [2], the overall prevalence of FMS was found to be 2.1% to 2.4% in women and 1.8% in men; however, the difference was not statistically significant [3]. Adequate treatment recommendations are therefore needed both in the interests of the welfare of the patient and for economic reasons. Current evidence-based guidelines are built on the fact that there is no single ideal treatment for FMS. Patient-tailored approaches are emphasized recommending non-pharmacological and pharmacological interventions according to individual symptoms (for example, pain, sleep problems, fatigue, and depression). Especially, self-management strategies (for example, exercise, psychological techniques) involving active patient participation should be an integral component of the therapeutic plan [4].

In this context, balneotherapy (BT) and hydrotherapy (HT) offer interesting treatment alternatives and are commonly used additional interventions in the management of FMS, despite ongoing debate about their effícacy. Prior research (an Internet survey of 2,596 people with FMS) found that around 26% of individuals suffering from FMS use pool therapy and 74% heat modalities (warm water, hot packs). The interventions perceived to be most effective (effectiveness rating ≥6.0) on a scale of 0 to 10, with 10 being most effective, were rest, (6.3 ± 2.5) (mean ± SD), heat modalities (6.3 ± 2.3), pain medication (6.3 ± 2.4), sleep medication (6.5 ± 2.7) and pool therapy (6.0 ± 3.0) [5].

However, the mechanisms by which immersion in mineral or thermal water or application of mud alleviates the symptoms of FMS are almost unknown. Pain, the key symptom of FMS, may be relieved by the hydrostatic pressure and the effects of temperature on the nerve endings, as well as by muscle relaxation [6]. Furthermore, it has been shown that thermal mud baths increase plasma levels of beta-endorphin, thus explaining their analgesic and antispastic effect, which is particularly important in patients with FMS [7]. The beneficial effects of water treatments are probably the result of a combination of specific (for example, buoyancy, aquatic resistance, heat) and unspecific effects (for example, change of environment, *spa-scenery*).

However, the definitions BT, HT and spa therapy are frequently confused and the terms tend to be used interchangeably [8]. In contrast to HT, which generally employs normal tap water, BT uses thermal mineral water from natural springs, but also natural gases (CO^2^, iodine, sulfur, radon, et cetera), peloids (mud) and other edaphic remedies (for example, hay) for medical treatment. BT is usually practiced in spas with their special therapeutic atmosphere as part of a complex therapy program, which is why the term is often used synonymously for spa therapy. Thalassotherapy is a special form of BT or spa treatment that uses seawater and the seaside climate. New definitions, such as health resort medicine, rather than BT and spa therapy, have not reached general acceptance [9].

Prior systematic reviews and meta-analyses covering BT (spa therapy) and HT in FMS have respectively covered the literature up to May 2011 [6], and December 2008 [10]. The systematic review by Terhorst *et al*. (2011) [11] on complementary and alternative medicine analyzed, among others, 11 studies on BT up to December 2010. The network meta-analysis by Nüesch *et al*. (2013) [12], which investigated pharmacological and non-pharmacological interventions (land- and water-based aerobic exercise, multicomponent treatment (MCT), BT and cognitive behavioral therapy (CBT)), covered the literature up to 2011. In summary, these reviews found some evidence of beneficial effects arising from BT and HT, however, due to methodological flaws, their efficacy remains unclear.

Despite these limitations, German and Israeli guidelines recommend temporary use of BT and HT (grade B/C) [13,14]. Furthermore, BT and HT are often part of MCT (at least one exercise and one psychological component) but they are not analyzed separately. In several evidenced-based guidelines and reviews, MCT and aerobic exercises (land-based or water-based) are strongly recommended [12-15]. The aim of the present review is to offer an update of the literature on BT and HT in FMS, with special focus on separate analyses of the different treatment modalities.

## Methods

This systematic review was performed according to the statement, *p*referred *r*eporting *i*tems for *s*ystematic *r*eviews and *m*eta-*a*nalyses (PRISMA) [16] and the recommendations of the Cochrane Collaboration [17].

### Literature search

Electronic bibliographic databases (Medline via Pubmed, Cochrane Central Register of Controlled Trials, EMBASE, and CAMBASE) were screened up to April 2013. The search strategy was constructed around a broad range of balneotherapeutic and hydrotherapeutic treatments: BT, HT, thalassotherapy, spa therapy, cryotherapy, thermotherapy, and phytothermotherapy combined with FMS. The search filter was restricted to randomized controlled trials (RCTs). Reference lists of relevant articles and reviews were examined for additional studies.

The search strategy for Pubmed was as follows: (“FMS” OR “fibromyal*”) AND “RCT” AND (“BT” OR “HT” OR “thalassotherapy” OR “spa therapy” OR “thermotherapy” OR “phytothermotherapy” OR “aquatic” OR “hydrogalvanic” OR “cryo” OR “pool exercise” OR “water-based” OR “pool-based” OR “stanger” OR “mud” OR “thermal water” OR “bath” OR “peloid” OR “natural therapeutic gas” OR “radon”). The search strategy applied a combination of text and keywords (medical subject heading (MeSH) terms) and was adapted for each database if necessary.

### Inclusion and exclusion criteria

*The criteria were as follows: 1) types of study:* RCTs were only eligible if they were published as full paper articles. No language restrictions were made; 2) *types of participants:* patients of any age diagnosed with FMS on recognized criteria were included; 3) *types of intervention:* studies that compared any kind of BT (mineral/thermal water, spa treatment, thalassotherapy, thermotherapy, peloids, natural therapeutic gas) or HT (treatment in plain water with or without exercise) with no treatment or any active treatment. Studies were excluded if BT/HT treatments were not the main intervention or if the intervention in treatment and control group were the same and only the co-therapies differed; and 4) *types of outcome:* studies assessing at least one symptom-specific outcome of the major FMS symptoms [18], such as pain (for example, tender point count (TPC), visual analog scale (VAS)), fatigue, sleep disturbances, depressive symptoms, health-related quality of life (HRQOL) and/or relevant pain-related psychological issues such as self-efficacy pain and/or objective tests of physical fitness, were included.

### Data extraction

The authors (JN, CS) of the review presented here independently extracted relevant study information (for example, participants, characteristics of the intervention and control, outcome measures, results) using predefined data fields, including risk-of-bias indicators. If necessary, existing inconsistencies were solved by discussion, and consensus achieved. For quantitative analysis the mean post-test values, or change scores when available, were used.

### Risk of bias assessment

The risk of bias for each study was determined independently by the same two authors (assessment of information in study reports) using the criteria of the Cochrane risk-of-bias tool. Disagreements were resolved by discussion to achieve consensus.

Summary assessment of risk-of-bias key domains (selection, performance, detection, attrition and reporting bias), was based on the three-tiered rating style as proposed by Higgins *et al*. [19]. Performance bias was not considered a key domain due to the required participatory nature of BT and HT. Studies with a high risk of bias in one of the key domains or unclear risk in at least two key domains were considered to be at high risk of bias. Studies with unclear risk in one of the key domains were considered to have unclear risk of bias. Only studies with low risk of bias in all key domains were graded as having low risk of bias. Analysis was done with the Review Manager (RevMan) version 5.2 risk-of-bias tool from the Cochrane Collaboration [21].

### Missing data

In the case of reported median, low and high end of range and sample size only, we estimated the mean and variance using the appropriate formula as mentioned by Hozo *et al*. [20].

### Data analysis and assessment of heterogeneity

RevMan version 5.2 [21] was used to analyze the data and perform testing of heterogeneity, using the *I*^2^ statistic, with the following categories: *I*^2^ = 25%, no heterogeneity; *I*^2^ = 50%, moderate heterogeneity; *I*^2^ = 75%, strong heterogeneity [22], and *P* ≤0.1 for the Chi^2^ test showing significant heterogeneity. We used Cohen’s categories to evaluate the magnitude of the effect size, calculated by standardized mean difference (SMD), with g >0.2 to 0.5, small effect size; g >0.5 to 0.8, medium effect size; and g >0.8, large effect size. We used the following modified levels of evidence descriptors to classify the results: (1) strong, if there were consistent findings among multiple (≥3) RCTs with low risk of bias; (2) moderate, if there were consistent findings among multiple high-risk RCTs and/or one low-risk RCT; (3) limited, with one high-risk RCT; (4) conflicting, with inconsistent findings among multiple RCTs; and (5) no evidence, no RCTs [23]. Whenever possible we used the results from intention-to-treat analysis. Negative SMDs indicate a beneficial effect of the experimental intervention.

### Subgroup and sensitivity analysis

Where at least two studies were available, subgroup analyses were pre-specified for different types of intervention. Additionally, control groups were compared (no treatment/active treatment). Waiting list or treatment-as-usual were classified as non-intervention control. The subgroup analyses were also used to examine potential sources of heterogeneity. Sensitivity analyses were performed for studies with high versus low risk of bias, respectively, for studies with serious flaws in one or more key domains and for sample size per treatment arm.

## Results

### Literature search

The literature search revealed 107 citations in accordance with the predefined search terms “FMS” and “BT” or “HT” and “RCT”. One additional study each was found in the reference lists of published reviews and the reference list of an already identified study: 52 duplicates were removed. A further 20 records were excluded because they did not fulfill the inclusion criteria (no papers on FMS and/or BT/HT (n = 7) [24-30]; different outcome measure (cost-effectiveness) (n = 2) [31,32]; reviews [33,34] (n = 2); no control group [35-38] (n = 4); not randomized [39-42] (n = 4); HT not the main treatment [43] (n = 1)).

Of the 37 articles that were assessed, four were excluded because of insufficient data reporting [7,44-46]. A further three studies were excluded because the main treatment (BT/HT) was the same both in the treatment and control group (Altan *et al*. [47]: baths in mineral water with and without exercise; Ammer and Melnizky [48]: whirl baths with and without etheric oils; Calandre *et al*. [49]: baths with two different kind of exercises). The remaining 30 articles included 2 reporting follow-up data to already included studies [50,51], and a further 4 reporting on the same study publication but with different outcome measures [52-55].

**Figure 1:**
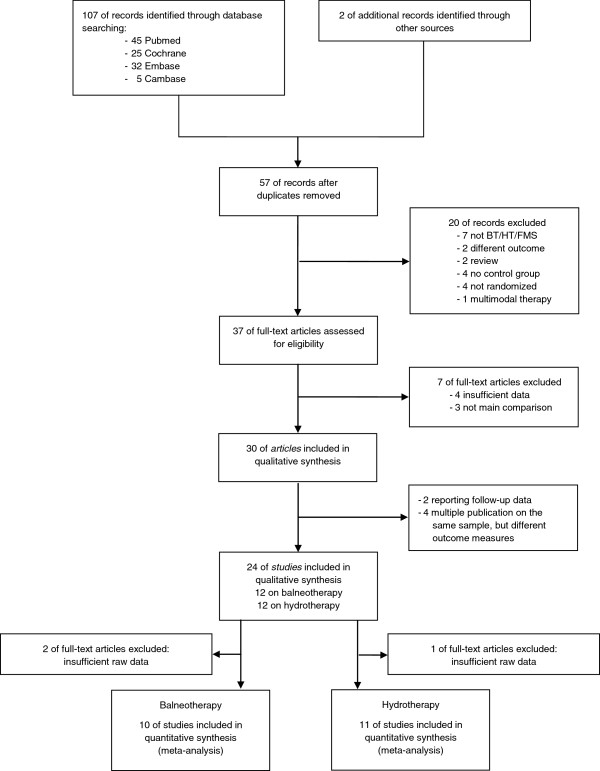
**Flowchart of the results of the literature search.** BT, balneotherapy; HT, hydrotherapy; FMS, fibromyalgia syndrome.

Finally, 24 studies met our inclusion criteria and were included in the qualitative analysis. Of these, 12 reported on HT [56-67] and 12 on BT [68-79]: 21 studies were suitable for quantitative analysis, 11 of which reported on HT and 10 on BT. Three studies had to be excluded from the quantitative analysis due to insufficient data reporting (HT: [59]; BT: [75,76]), (see Figure 1).

### Description of included trials

The characteristics of the included studies are detailed in the following tables (see Additional files 1 and 2). The studies were separated according to treatment modalities: Additional file 1: HT with the subgroups, HT with exercise (n = 10) and hydrogalvanic (Stanger) bath (n = 2). Additional file 2: BT with the subgroups mineral water (n = 3), spa therapy (n = 3), sulfur bath (n = 2), thalassotherapy (n = 1), phytothermotherapy (n = 1), mud (n = 1), acratothermal water (n = 1). Study characteristics for all trials included in qualitative synthesis are summarized below.

### Patient characteristics

Participants’ age across the studies ranged from 18 to 73 years. The median of the mean age in the treatment group was 45.2 years compared to 46.3 years in the control group. Disease duration was reported in 19 studies and ranged from 1.3 to 24.0 years. The median of mean disease duration was 8.4 years. Over 96% of the participants were women. Sixteen studies involved women only [56,57,62-71,73,75-77], and eight studies included both women and men [58-61,72,74,78,79]. The median of the mean pain baseline values reported in 20 studies was 7.1 (5.5 to 9.1). Pain scores were assessed in the four remaining studies but not reported separately [57,73,74,76].

### Study characteristics

#### Origin of studies

Two RCTs originated from Canada [59,60], eight from Turkey [57,58,69,71,72,75,77,78], two from Brazil [56,68], one from Israel (Dead sea) [70], two from Italy [73,74], one from Austria [61], three from Spain [62,66,67], one from Norway [63], two from Sweden [64,65], one from Germany [76] and one from The Netherlands [79].

#### Setting

Eighteen studies were conducted in outpatient settings [56,58-69,72-74,76,78] and six in inpatient settings [57,70,71,75,77,79]. Seven studies were conducted within spa resorts either with in-patients or outpatients [69-71,73,74,77,79]. Patients were referred from primary, secondary and tertiary care settings.

#### Inclusion and exclusion criteria

In all the studies, FMS was diagnosed according to the ACR criteria [80]. Patients with severe diseases were excluded in 18 studies [56-58,60,62-69,72-76,79] and patients with mental disorders and/or on antidepressant drugs in 11 studies [57,58,60,62,64-67,72,75,76]; 6 studies didn’t report exclusion criteria [59,61,70,71,77,78].

#### Reporting of adverse events

Adverse events were reported in four studies [56,65,68,79]. In all cases the adverse events were not indicated as a cause of interruption or dropouts. Seven studies [57,58,72-75,78] clearly reported that there were no adverse events. The remaining 13 studies gave no information on adverse events. No serious adverse events were reported (for details see Additional files 1 and 2).

### Intervention characteristics

HT interventions used, among others, hydrogalvanic/Stanger bath in two studies [57,61] and exercises in plain water [56,58-60,62-67]. BT interventions were spa therapy [71,77,79], thalassotherapy [68], phytothermotherapy [73], mud [74], sulfur bath [70,76], baths in mineral/thermal water [69,72,78] and in acratothermal water [75]. Treatment duration in the HT group ranged from 5.0 to 32.0 weeks, with a median of 15.5 weeks, in contrast to BT studies with shorter duration ranging from 1.5 to 12.0 weeks and a median of 2.0 weeks. Median follow-up duration was similar for both HT and BT at 2.5 and 3.5 months respectively.

### Outcome measures

Different VAS were used to measure pain. Four studies did not report how pain was measured [57,73,74,76]. Twenty studies used the Fibromyalgia Impact Questionnaire (FIQ) to measure HRQOL. Nine studies [56,58,60,68,69,71,72,75,79] measured depressed mood by the Beck Depression Inventory (BDI).

### Risk of bias

Only 5 of the 24 studies included had low risk of bias [56,57,64,67,68]; a further 5 were assigned as having unclear risk (studies with one unclear judgement; unclear allocation: [65,71,77]; selective reporting: [66]; unclear outcome assessment blinding: [79]). The remaining 14 studies were at high risk of bias, as they had two or more unclear judgements in the key domains, including 5 studies with serious flaws in one or more key domains [59,60,63,75,76]. For details see categorization of risk of bias at the individual study level (see Additional file 3).

#### Sequence generation and treatment allocation

Of 24 studies, 10 had unclear risk of selection bias in both domains, 2 were considered to be at high risk because of serious randomization flaws [59,75]. Half the studies reported adequate randomization, but only seven adequate allocation concealment [4,6,21,47,52,69,80].

#### Similar baseline

All studies had low risk of selection bias with the exception of two, one with unclear risk (unclear reporting; [76]) and one with high risk due to significant differences in baseline characteristics in a major FMS symptom (TPC) [63]).

#### Blinding of participants and personnel

Performance bias was not considered a key domain. Due to the participatory nature of BT and HT blinding is not feasible.

#### Incomplete outcome data

Of the 24 studies, 19 were assigned low risk of attrition bias (criteria: attrition rate reported, not exceeding 20% or intention-to-treat analysis). Five studies were assigned unclear or high risk of bias because two had high dropout rates [60,63] (high risk of bias) and the dropout rate was not clearly reported in three studies [70,76,78].

#### Selective reporting

Two studies were assigned high risk of bias [59,75]; thus, reporting was insufficient and not in alignment with the values presented in tables. A further five had unclear risk of reporting bias due either to double reporting [66,70] or incomplete/inconsistent outcome reporting [60,61,63].

#### Blinding of outcome assessment

Fifteen of the 24 studies had low risk of detection bias for outcome assessment, eight had unclear risk [58,59,61,62,69,72,75,79], and one was assigned a high risk of bias [76] (see Additional file 4).

### Subgroup analyses

#### Hydrotherapy

**Figure 2:**
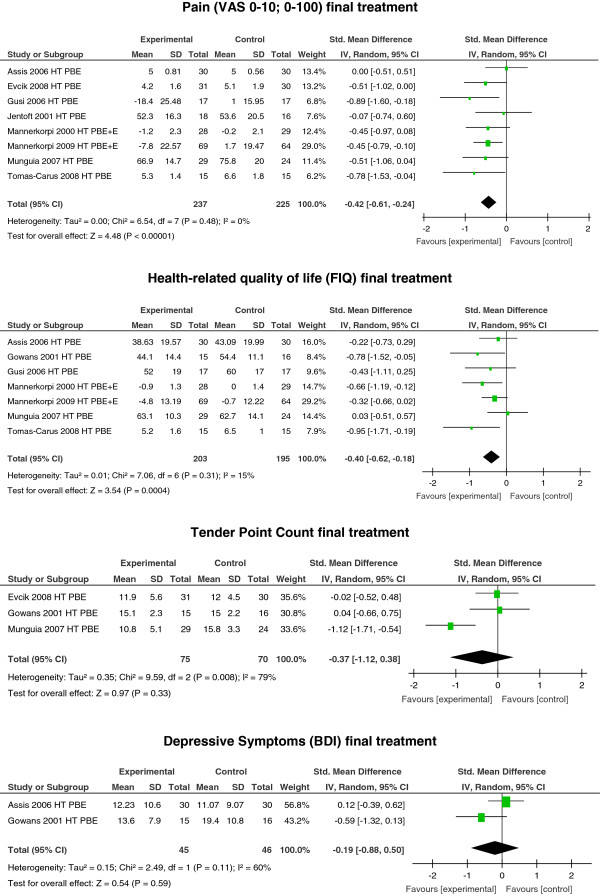
**Treatment effects of exercises in plain water (hydrotherapy, HT) at the end of treatment.** PBE, pool-based exercise; +E, plus education; VAS, visual analog scale; FIQ, fibromyalgia impact questionnaire; BDI, Beck depression inventory.

Meta-analyses showed moderate-to-strong evidence (consistent findings among multiple (≥3) RCTs with low risk of bias) for a small reduction in pain with exercises (pool-based exercise (PBE) in plain water (HT) at the end of treatment; SMD −0.42; 95% CI −0.61, −0.24; *P* <0.00001; *I*^2^ = 0% (eight studies: three low-risk studies [56,64,67], two unclear-risk studies [65,66], three high-risk studies [58,62,63]). Concerning HRQOL (FIQ) at the end of treatment, there was moderate-to-strong evidence for a small improvement; SMD −0.40; 95% CI −0.62, −0.18; *P* = 0.0004; *I*^2^ = 15% (seven studies: three low-risk studies [56,64,67], two unclear-risk studies [65,66], two high-risk studies [60,62]). For depressive symptoms (BDI) and TPC no significant effect was seen at the end of treatment (BDI: SMD −0.19; 95% CI −0.88, 0.50; *P* = 0.59; *I*^2^ = 60% (one low-risk [56] and one high-risk study [60]); TPC: SMD −0.37; 95% CI −1.12, 0.38; *P* = 0.33; *I*^2^ = 79% (one unclear risk [66] and two high-risk studies [58,60]) (see Figure 2).

#### Comparison group

Subgroup analysis of the type of comparison group suggests that RCTs comparing HT to no treatment (usual care) or other types of active control had a significant effect, but not when compared to land-based exercise (see Additional file 5).

#### Balneotherapy

**Figure 3:**
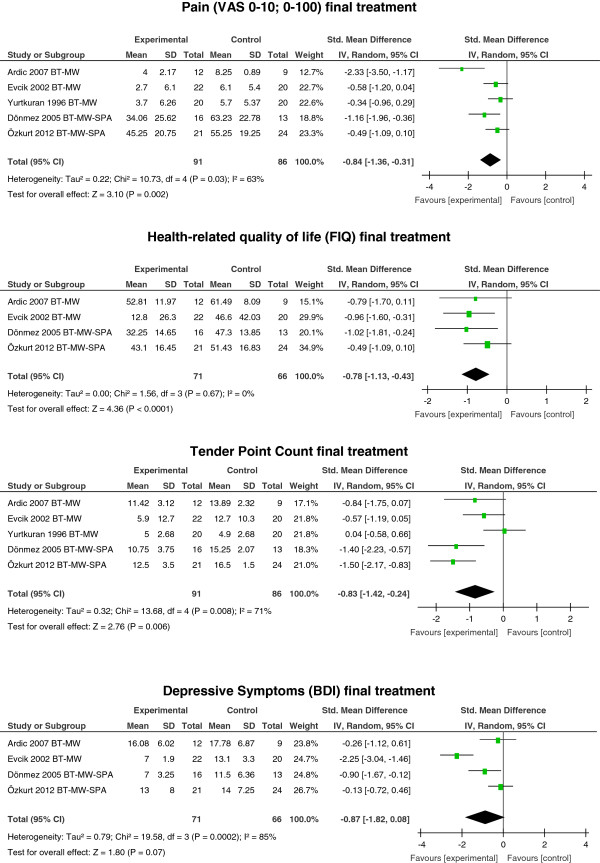
**Treatment effects of balneotherapy (BT) in mineral/thermal water (MW), within a spa center (SPA) or not, at the end of treatment.** VAS, visual analog scale; FIQ, fibromyalgia impact questionnaire; BDI, Beck depression inventory.

Meta-analyses showed moderate evidence for a large reduction of pain at the end of treatment with BT in mineral/thermal water, regardless of whether within a spa center (SPA) or not: SMD −0.84; 95% CI −1.36, −0.31; *P* = 0.002; *I*^2^ = 63% (five studies: two unclear-risk studies [71,77] and three high-risk studies [69,72,78]). Moderate evidence was seen for a medium improvement in HRQOL (FIQ); SMD −0.78; 95% CI −1.13, −0.43; *P* <0.0001; *I*^2^ = 0% (four studies: two unclear-risk [71,77] and two high-risk studies [69,72]). Moderate evidence for a large improvement was seen for TPC: SMD −0.83; 95% CI −1.42, −0.24; *P* = 0.006; *I*^2^ = 71% (five studies: two unclear-risk [71,77] and three high-risk studies [69,72,78]). There was no significant effect on depressive symptoms (BDI) at the end of treatment (SMD −0.87 −1.82, 0.08; *P* = 0.07; *I*^2^ = 85% (four studies: two unclear-risk [71,77] and two high-risk studies [69,72]) (see Figure 3).

### Follow up

**Figure 4:**
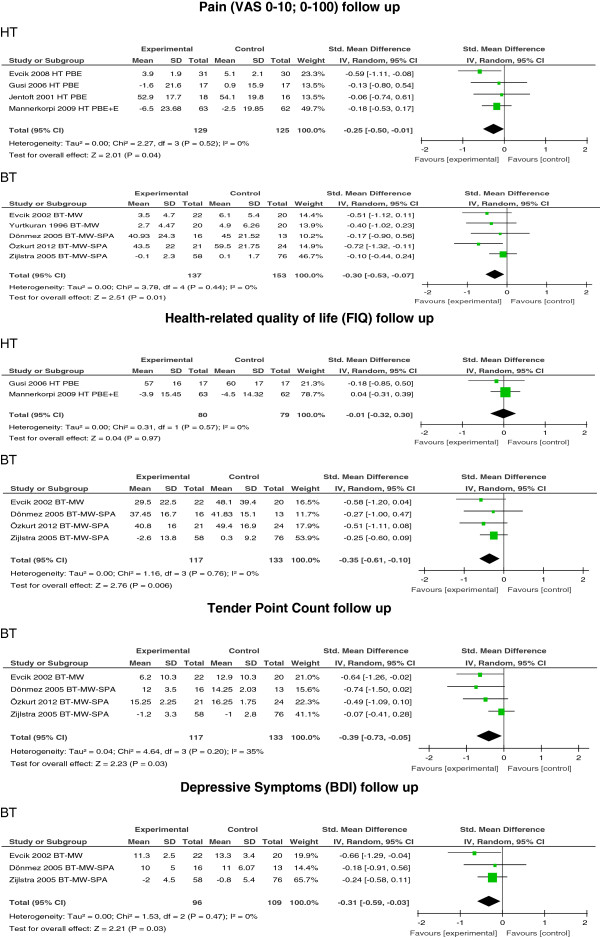
**Treatment effect of hydrotherapy (HT)/balneotherapy (BT) at follow up.** VAS, visual analog scale; FIQ, fibromyalgia impact questionnaire; BDI, Beck depression inventory.

Findings at follow up showed that a small reduction of pain was maintained for HT and BT: SMD −0.25; 95% CI −0.50, −0.01; *P* = 0.04; *I*^2^ = 0% for HT and SMD −0.30; 95% CI −0.53, −0.07; *P* = 0.01; *I*^2^ = 0% for BT. Only BT showed significant results for HRQOL (FIQ) (SMD −0.35; 95% CI −0.61, −0.10; *P* = 0.006; *I*^2^ = 0%). With regard to TPC and BDI, only BT studies provided follow-up data with SMD −0.39; 95% CI −0.73, −0.05; *P* = 0.03; *I*^2^ = 35% for TPC and SMD −0.31; 95% CI −0.59, −0.03; *P* = 0.03; *I*^2^ = 0% for BDI (see Figure 4).

### Analysis of overall effects

Taking into account all available studies, regardless of treatment modality, meta-analysis provided moderate evidence for a medium reduction of pain at the end of treatment; SMD −0.57; 95% CI −0.77, −0.38; *P* <0.00001; *I*^2^ = 45%. Results are shown for HT, BT and diverse treatments: hydrogalvanic bath (Stanger), mud therapy, sulfur bath and thalassotherapy (see Additional file 6).

### Sensitivity analyses

Sensitivity analysis according to potential risks of bias showed no significant difference between the effect size of pain (HT) at the end of treatment and risk of bias (see Additional file 7). Analysis according to sample size (<25, >25) shows a slightly larger effect size and broader CIs in small studies (*P* = 0.54) (see Additional file 8).

Statistical heterogeneity of analysis for the effect size of pain in the BT group (*I*^2^ = 63%) was substantially decreased (*I*^2^ = 0%) by removing the study of Ardiç *et al*. [69] (pharmacological co-therapies not allowed; non-intervention control group). The magnitude of the effect size was decreased to SMD −0.58; 95% CI −0.91, −0.26, *P* = 0.0004, corresponding to a medium effect.

### Publication bias

Visual analysis of the funnel plot shows a symmetric picture, with one outlier study already identified by sensitivity analysis [69]. This indicates that the results of the meta-analysis can be regarded as robust against potential reporting bias (see Additional file 9).

## Discussion

### Summary of evidence

The primary aim of this systematic review and meta-analysis was to determine the therapeutic benefit of BT and HT in the management of FMS, with special focus on separate analyses of the different treatment modalities. For HT with exercise we found moderate-to-strong evidence (consistent findings among ≥3 RCTs with low risk of bias) for a small improvement in pain (eight studies, 462 participants; including three low-risk studies, 223 participants) and HRQOL (seven studies, 398 participants; including three low-risk studies, 223 participants). Follow-up data provided moderate evidence (consistent findings among multiple high-risk RCTs and/or one low-risk RCT) for maintenance of improvement, at least with regard to pain (four studies, 254 participants; including one low-risk study, 125 participants). However, no evidence was found for improvement of depressive symptoms (BDI) and TPC. Furthermore, no group difference was found when comparing water-based exercise to land-based exercise. This is in accordance with the review by Häuser *et al*. from 2010 [81].

We found moderate evidence of a medium-to-large effect on pain and TPC for BT with mineral/thermal water (five studies, 177 participants; including three high-risk and two unclear-risk studies), a medium effect on HRQOL, and no significant effect on depressive symptoms (BDI). Moderate evidence for maintenance of these improvements was found at follow up. However, the effects were smaller. The results confirm the conclusions of other reviews on BT [6,82].

Besides these two larger groups, further subgroup analyses were not possible due to the limited number of available studies and/or provided data. This is also true of the follow-up data provided, where only a few studies remained for statistical analyses. The evidence on the long-term effects that can be concluded from this meta-analysis is limited.

No conclusions can be drawn on hydrogalvanic/Stanger baths, thalassotherapy, mud baths, phytothermotherapy or sulfur baths, which were only represented by one study each. So as not to lose the information provided by these studies, we pooled all the available studies in an overall analysis, which showed similar effects (reduction of pain) to HT or BT.

Concerning safety, only preliminary conclusions can be drawn, because reporting of adverse events and the reasons for dropouts was poor. The data suggest that HT and BT are safe and well-accepted treatments, which is in line with other recommendations [10,83], and we should not forget the daily experience of patients and the general population practising some kind of BT or HT.

Male participants were rarely included in the study populations, and separate gender comparisons were not reported. Evidence for treatment effects in the management of FMS in men is limited. Furthermore, it has to be taken into account that the population of FMS patients participating in a trial is selected. Generalisability may be restricted [84].

### Limitations

As so often in evidence-based approaches to nonpharmacological modalities, limitations are inherent and inevitable. This is especially true for BT, which depends on local conditions such as climate or water composition and provides a large variety of treatment modalities. Absence of blinding is also inevitable wherever treatment requires active participation on the part of the study subjects and clinicians.

There are also several methodological limitations. The analyses were underpowered due to the small number of studies and patients included. Analysis according to sample size (<25, >25) showed a slightly larger effect size and broader CIs in small studies (*P* = 0.54). The methodological quality (risk of bias) of the included studies varied, and was slightly better in HT studies than BT studies. Although some studies had low risk of bias, the majority - especially older studies - were associated with unclear or high risk of bias. Nevertheless, sensitivity analyses could show, at least in HT studies, that the effect sizes were not affected by methodological bias. Due to the limited number of BT studies, sensitivity analyses could not be performed here. Furthermore, the sample sizes in the BT studies were very small (<25 per treatment arm), except for one study [79]. Unfortunately, in this study, no results were collected for the control group after treatment. Thus, the data were not analyzed and only follow-up data were used.

Heterogeneity was not present in the HT studies, in contrast to considerable heterogeneity in the BT studies. This could be explained by the fact that co-therapies were not allowed in one study, which also had a non-interventional control group [69]. As far as selection bias is concerned, it is not possible to assess the extent to which the results may be influenced. Most of the studies reported unclear randomization methods as well as insufficient allocation concealment. The studies that allowed co-therapies did not control their effects for dosage or changes in concomitant therapies.

A strength of this review is the homogenous pool of treatment approaches selected for subgroup analyses, based on the professional expertise in the field of balneology of one of the authors (JN). The evidence of the integrated effect sizes seems robust, especially since publication bias is not plausible after visual analysis of the funnel plot, showing a symmetric picture, except for one outlier study [69] already identified by sensitivity analysis. Commencing from a systematic and thorough search of the literature (CS) we are confident not to have missed any larger important study.

## Conclusions

In summary, based on the limited number of studies analyzed, small sample sizes and risk of bias attributed to the studies, it appears difficult to determine the overall benefit of BT and HT. There is a risk of overestimating the evidence on the efficacy of HT and even more so BT. However, although evidence is limited, recommendations in recent evidence-based interdisciplinary guidelines emphasize a patient-tailored approach with aerobic exercises, CBT and MCT according to the key symptoms of FMS [4]. In this context, BT and HT offer a wide variety of treatment opportunities, which can be perfectly adapted to the patients’ abilities and preferences. Unlike pharmacological treatments with questionable clinical relevance and frequent side effects [12], the results of this review underline the potential value of BT and HT as supplementary therapy in the management of major symptoms of FMS.

In order to provide a better database for meta-analyses (internal validity), the use of a core set of outcome measures (outcome measures in rheumatology (OMERACT) [85]) including response rates is desirable. Future authors should use the consolidated standards of reporting trials (CONSORT) checklist [86] to report study results. Major interest should focus on long-term results and maintenance of beneficial effects. Given the popularity of BT and HT among patients with FMS, further studies with robust methodology are warranted to demonstrate and confirm the therapeutic benefits.

## Abbreviations

ACR: American College of Rheumatology; BDI: Beck depression inventory; BT: balneotherapy; CBT: cognitive behavioral therapy; CWP: chronic widespread pain; DWR: deep-water-running; FIQ: fibromyalgia impact questionnaire; FMS: fibromyalgia syndrome; HRQOL: health-related quality of life; HT: hydrotherapy; LBE: land-based exercise; MCT: multicomponent treatment; MW: mineral water; PBE: pool-based exercise; RCT: randomized controlled trial; SB: sulfur bath; SMD: standardized mean difference; SPA: spa center; TPC: tender point count; TT: thalassotherapy; VAS: visual analog scale.

## Competing interests

JN receives support from balneology organisations such as *Deutscher Heilbäderverband* and *Heilbäderverband Baden-Württemberg*, and is member of these organisations. None of these organisations financed this work. The authors declare that they have no competing interests.

## Authors’ contributions

JN carried out the study concept and design, participated in the interpretation of the data, and helped draft the manuscript. CS carried out the statistical analysis, participated in drafting of the manuscript and interpretation of the data. Both authors read and approved the final manuscript.

## Supplementary Material

Additional file 1: Table S1 Hydrotherapy - characteristics of the included studies. Hydrotherapy with the subgroups, hydrotherapy (HT) with exercise (n = 10) and hydrogalvanic (Stanger) bath (n = 2). Detailed study characteristics: author, year, risk of bias (high, unclear, low), intent-to-treat analysis (yes/no), sample size (treatment group/control group), sex, mean age, fibromyalgia syndrome (FMS) (duration/years), pain (visual analog scale, VAS), dropouts (n), treatment (treatment group/control group), co-therapies, outcome measures (primary/secondary outcome), treatment efficacy and safety (adverse effects). Click here for file

Additional file 2: Table S2 Balneotherapy - characteristics of the included studies. Balneotherapy with the subgroups, mineral water (n = 3), spa therapy (n = 3), sulfur bath (n = 2), thalassotherapy (n = 1), phytothermotherapy (n = 1), mud (n = 1), acratothermal water (n = 1). Detailed study characteristics: author, year, risk of bias (high, unclear, low), intent-to-treat analysis (yes/no), sample size (treatment group/control group), sex, mean age, fibromyalgia syndrome (FMS) (duration/years), pain (visual analog scale, VAS), dropouts (n), treatment (treatment group/control group), co-therapies, outcome measures (primary/secondary outcome), treatment efficacy and safety (adverse effects). Click here for file

Additional file 3 **Risk of bias summary.** The file contains authors’ judgements about each risk-of-bias item for each included study. Risk of bias: high, unclear, low. Items: selection bias (random sequence generation, allocation concealment, similar baseline characteristics); performance bias (blinding of participants and personnel); attrition bias (incomplete outcome data); reporting bias (selective reporting); detection bias (blinding of outcome assessment). BT, balneotherapy; HT, hydrotherapy; MW, mineral water; PBE, pool-based exercise; SB, sulfur bath; TT, thalassotherapy; Spa, spa center; Stanger, Stanger bath; Mud, mud bath; Hay, phytothermotherapy; PBE + E, pool-based exercise + education; PTM, physical therapy modalities (transcutaneous electrical nerve stimulation (TENS), ultrasound, infrared). Click here for file

Additional file 4 **Risk of bias graph.** The file contains authors’ judgement of each risk of bias item presented as percentages across all included studies. Click here for file

Additional file 5 **Subgroup analysis for control group (LBE = land-based exercise; PBE = pool-based exercise; +E = education).** The file contains the subgroup analysis regarding type of comparison group. Click here for file

Additional file 6 **Treatment effect of hydrotherapy (HT), balneotherapy (BT) and diverse therapies (hydrogalvanic bath (Stanger), mud therapy, sulfur bath (SB) and thalassotherapy (TT) on pain.** The file contains the analysis of overall effects, taking into account all available studies, regardless of treatment modality. Click here for file

Additional file 7 **Sensitivity analysis for risk of bias (hydrotherapy (HT), pain).** The file contains the forest plot displaying the relationship between effect size and risk of bias. Click here for file

Additional file 8 **Sensitivity analysis for sample size (hydrotherapy (HT), pain).** The file contains the forest plot displaying the relationship between effect size and sample size. Click here for file

Additional file 9 **Funnel plot (based on data of overall analysis, n = 17 studies).** The file contains the scatter plot of the intervention effect estimates (SMD) from individual studies against their standard errors (SE). Publication bias may lead to asymmetry in funnel plots on visual inspection. Click here for file
