# Supplementary material for: Therapeutic benefit of balneotherapy and hydrotherapy in the management of fibromyalgia syndrome: a qualitative systematic review and meta-analysis of randomized controlled trials
Source: Arthritis Res Ther. 2014 Jul 7;16(4):R141. doi: 10.1186/ar4603 (PMC4227103; doi:10.1186/ar4603)
Supplement: Additional file 1: Table S1 — Hydrotherapy - characteristics of the included studies. Hydrotherapy with the subgroups, hydrotherapy (HT) with exercise (n = 10) and hydrogalvanic (Stanger) bath (n = 2). Detailed study characteristics: author, year, risk of bias (high, unclear, low), intent-to-treat analysis (yes/no), sample size (treatment group/control group), sex, mean age, fibromyalgia syndrome (FMS) (duration/years), pain (visual analog scale, VAS), dropouts (n), treatment (treatment group/control group), co-therapies, outcome measures (primary/secondary outcome), treatment efficacy and safety (adverse effects). [file ar4603-S1.docx]

**TABLE 1: Hydrotherapy - characteristics of the included studies.**

| Studies  Author, year | Risk of bias  ROB:  high/  unclear/  low  ITT  Intent- to-treat analysis  yes/no | Treatment  duration/  Follow-up  T: treatment  FU: follow-up  Setting, country  Inpatients/  outpatients | Sample  size  Treatment  group/  Control  group  Sex  Mean age  FMS: Duration/  years  Pain (VAS) | Dropouts  NR:  not  reported | Treatment  Treatment group (TG)  Control group (CG)  CT: Co-therapies, NM: not monitored | Outcome measures  FIQ: Fibromyalgia Impact Quest.  BDI: Becks Depr. Scale  VAS: Visual Analogue Scale  TPC: Tender Point Count  I. Primary outcome  II. Secondary outcome | Treatment efficacy and safety  AE: Adverse effects  NR: not reported |
| --- | --- | --- | --- | --- | --- | --- | --- |
| **Hydrotherapy and exercise** | | | | | | | |
| Assis et al.  2006 [56] | ROB:  low  ITT: yes | T: 15 weeks  (3 days/week)  FU: no  University,  Brazil outpatients | 30/30  women  age: 43.4/42.2  FMS 5.2 y  Pain 8.0 | 4/4 | TG: pool-based exercise/ deep water running (PBE); (28-31°C), 60 min  CG: land-based exercise (LBE),  60 min  CT: medication continued, acetaminophen allowed; NM | I. Pain (VAS)  II. FIQ  II. BDI  II. SF-36  II. PGA  II. physical fitness | No sign. difference between groups (all outcomes)  AE: TG (10): muscle pain (4), tinea pedis (1); CG (16): muscle pain (12), impingement syndrome (1), bilateral ankle arthritis (1), Baker cyst (1)  No SAE, no dropouts because of AE |
| Evcik et al.  2008 [58] | ROB:  high  ITT: no | T: 5 weeks  (3 days/week)  FU: 6 months  University, Turkey outpatients | 33/30  60 w/1 m  age: 43.8/42.8  FMS 3 y  Pain 6.2 | 2/0 | TG: aquatic exercise program (33°C) 60 min  CG: home-based exercise program, 60 min.  CT: Antidepressive drugs or NSAIDs **not** allowed; NM | Pain (VAS)  FIQ  TPC  BDI  I./II. NR | Sign. improvement for pain in favour of TG after treatment and at FU (12 and 24 weeks).  No sign. difference between groups for other outcomes  AE: none |
| Gowans et al.  1999 [59] | ROB:  high  ITT: no | T: 6 weeks  (2 days/week)  FU: 3 months  (only TG)  Canada outpatients | 23/22  32 w / 9 m  age: 44.3/46.6  FMS NR  Pain 7.5 | 3/1 | TG: Pool exercise (warm water, °C NR) 30 min (aerobic endurance, flexibility exercises),  patient education, 1h  CG: no treatment (waiting list)  no CG for FU at 3 months  CT: Medication use at baseline: FMS related drugs, e.g. NSAIDs, narcotics; NM | 6MWT  FIQ  ASES  Patients’ knowledge of fibromyalgia management  I./II. NR | Sign. improvement in 6MWT and 2 subscales of FIQ (Feel bad, Fatigue morning) in favour of TG    FU 3 months: sign. improved vs. baseline for 6MWT and 2 subscales of FIQ (Feel bad, fatigue morning) (No CG for FU)  AE: NR |

| **TABLE 1: Hydrotherapy and exercise continued** | | | | | | | |
| --- | --- | --- | --- | --- | --- | --- | --- |
| Studies  Author, year | Risk of bias  ROB:  high/  unclear/  low  ITT  Intent- to-treat analysis  yes/no | Treatment  duration/  Follow-up  T: treatment  FU: follow-up  Setting, country  Inpatients/  outpatients | Sample  size  Treatment  group/  Control  group  Sex  Mean age  FMS: Duration/  years  Pain (VAS) | Dropouts  NR:  not  reported | Treatment  Treatment group (TG)  Control group (CG)  CT: Co-therapies, NM: not monitored | Outcome measures  FIQ: Fibromyalgia Impact Quest.  BDI: Becks Depr. Scale  VAS: Visual Analogue Scale  TPC: Tender Point Count  I. Primary outcome  II. Secondary outcome | Treatment efficacy and safety  AE: Adverse effects  NR: not reported |
| Gowans et al. 2001 [60], 2004 [50] | ROB:  high  ITT: unclear | T: 23 weeks  (3 days/week)  FU: 6/12 months (only TG)  Hospital, Canada outpatients | 27/23  44 w / 6 m  age: 49.1/46.7  FMS 2.8 y  Pain  50.6 (ASES: 0-100) | 12/7  (38%) | TG: stretching and aerobic exercise in warm water (°C NR), 30 min  CG: no treatment  CT: not allowed to start aerobic exercise, change mood-altering medication or seek professional treatment for mood disturbances  Medication as FMS related drugs, e.g. NSAIDs, narcotics, etc. continued; NM | I. 6MWT  I. BDI  II. FIQ  II. ASES  II. STAI  II. MHI | Sign. improvement in 6MWT and BDI, and FIQ, ASES, STAI and 3 subscales of MHI in favour of TG    FU-12months: Sign. improvement vs. baseline for 6MWT and BDI, and FIQ, STAI and 2 subscales of ASES (No CG for FU)  AE: NR |
| Gusi et al.  2006 [62]  Tomas-Carus et al.  2007 [55] | ROB:  high  ITT: no | T: 12 weeks  (3 days/week)  FU: 3 months  University, Spain  outpatients | 18/17  women  age: 51/51  FMS 22 y  Pain 63.1  (0-100) | 1/0 | TG: Pool exercise, 60 min (33°C)  (stretching, aerobic exercise, mobility & lower limb strengthening exercises)  CG: no treatment (normal activity continued, without any exercise!)  **FU: De-training period, both groups were instructed to avoid physical exercises**  CT: NR | I. Isokinetic muscle strength  I. HRQOL/EQ-5D  I. Pain VAS (0-100)  II. div. questionnaires on spare time/work activities  SF-36  FIQ  Canadian Aerobic Fitness  1-leg stance  I./II. NR | TG: Sign. improvement for pain and EQ-5D and 7 of 8 subscales of SF36 and 4 of 7 fitness tests in favour of TG at week 12 and 3 subscales of EQ50 and 2 of 8 subscales of SF36 at week 24.  AE: NR |

| **TABLE 1: Hydrotherapy and exercise continued** | | | | | | | |
| --- | --- | --- | --- | --- | --- | --- | --- |
| Studies  Author, year | Risk of bias  ROB:  high/  unclear/  low  ITT  Intent- to-treat analysis  yes/no | Treatment  duration/  Follow-up  T: treatment  FU: follow-up  Setting, country  Inpatients/  outpatients | Sample  size  Treatment  group/  Control  group  Sex  Mean age  FMS: Duration/  years  Pain (VAS) | Dropouts  NR:  not  reported | Treatment  Treatment group (TG)  Control group (CG)  CT: Co-therapies, NM: not monitored | Outcome measures  FIQ: Fibromyalgia Impact Quest.  BDI: Becks Depr. Scale  VAS: Visual Analogue Scale  TPC: Tender Point Count  I. Primary outcome  II. Secondary outcome | Treatment efficacy and safety  AE: Adverse effects  NR: not reported |
| Jentoft et al.  2001 [63] | ROB:  high  ITT: no | T: 5 months  (2 days/week)  FU: 6 months  Hospital, Norway outpatients | 22/22  women  age: 42.9/39.4  FMS 11.1 y  Pain 6.9 | 4/6 | TG: Pool-based exercise (PBE), 60 min (34°C)  (modified Norwegian aerobic)  CG: land-based exercise (LBE), 60 min  (original Norwegian aerobic)  CT: NR | Pain (VAS)  FIQ  TPC  ASES  I./II. NR | No sign. difference between groups, except for improved grip strength in favour of CG at week 20.  AE: NR |
| Manner-korpi et al.  2000 [65]  Manner-korpi et al.  2002 [51] | ROB:  unclear  ITT: no | T: 6 months  (1 day/week)  FU: 6/24 months (only TG)  Primary health care and rheumatology clinics,  University, Sweden outpatients | 37/32  women  age: 47/45  FMS 8.4 y  Pain 7.8  For FU: 28/0  women | 9/3  2/0 | TG: Pool exercise, 35 min  (endurance, flexibility, co-ordination and relaxation);  education sessions (6 x 1 h) focusing on coping strategies, importance of physical activity  CG: no treatment (usual activities continued)  CT: Baseline medication continued without change;  Patients (79%) reported self initiated changes in lifestyle. | I. FIQ (total score)  I. 6MWT  II. FIQ (subscales)  SF-36  MPI-S*  ASES-S*  AIMS  QoL-S*  Grippit measure  Chair test (evaluation of lower extremity endurance by recording the number of repetitive and fast movements of sitting down and standing up from a chair in 1 min)  * ‘S’ indicates validated swedish version | Sign. improvement in 6MWT, FIQ total score, 2 of 10 FIQ subscales, 2 of 9 further functional tests, 2 of 8 subscales of SF 36, 2 of 8 subscales of MPI-S, QOLS and AIMS in favour of TG  AE: deterioration of shoulder function in TG and CG, sign. less in TG.  FU-6 months: sign. improved vs. baseline for 6MWT, FIQ total score, 5 of 10 FIQ subscales, 3 of 4 further functional tests, 3 of 8 subscales of SF-36  FU 24 months: sign. improved vs. baseline for 6MWT, 4 of 10 FIQ subscales, 2 of 4 further functional tests, 3 of 8 subscales of SF-36 |

| **TABLE 1: Hydrotherapy and exercise continued** | | | | | | | |
| --- | --- | --- | --- | --- | --- | --- | --- |
| Studies  Author, year | Risk of bias  ROB:  high/  unclear/  low  ITT  Intent- to-treat analysis  yes/no | Treatment  duration/  Follow-up  T: treatment  FU: follow-up  Setting, country  Inpatients/  outpatients | Sample  size  Treatment  group/  Control  group  Sex  Mean age  FMS: Duration/  years  Pain (VAS) | Dropouts  NR:  not  reported | Treatment  Treatment group (TG)  Control group (CG)  CT: Co-therapies, NM: not monitored | Outcome measures  FIQ: Fibromyalgia Impact Quest.  BDI: Becks Depr. Scale  VAS: Visual Analogue Scale  TPC: Tender Point Count  I. Primary outcome  II. Secondary outcome | Treatment efficacy and safety  AE: Adverse effects  NR: not reported |
| Manner-korpi et al.  2009 [64] | ROB:  low  ITT: yes | 5 months  (1day/week)  FU 11-12 months  University, Sweden outpatients | 81/85*  women  age: 44.6/46.5  FMS 10.3 y  Pain 67.7  (FIQ)  *Patients: FMS n = 134  Chr. wide-spread pain  n = 32 | 6/8 | TG: pool-exercise (33°C) 45 min., 20 sessions; education programme (1h), 6 sessions  CG: education programme  Pharmacological treatment: analgesics, psychotropics  CT: medication continued and monitored | I.FIQ  I.6MWT  II. Pain (FIQ)  II. Fatigue (FIQ)  II. SF-36  II. Depression (HADS)  LTPAI (leisure time)  SCI (stress)  MFI (fatigue) | Sign. improvement in FIQ total score, FIQ pain, 1 MFI subscale in favour of TG  FU-12 months: TG: sign. improved vs. baseline 6MWT, 1 of 6 subscales of SF 36, SCI  CG sign. improved vs baseline for FIQ total, FIQ pain, 1 MFI subscale  AE: NR |
| Munguia-Izquierdo and Legaz-Arrese  2007 [66], 2008 [52] | ROB:  unclear  ITT: yes | T: 4 months  (3 days/week)  FU: no*  University, Spain outpatients | 34/24  women  age: 50/46  FMS 14 y  Pain  66.9 (FIQ) | 5/0 | TG: aquatic training (32°C) 60 min.  CG: no treatment  CT: medication continued; NM | Pain (VAS 0-100)  FIQ  TPC  PSQI (sleep)  STAI  PASAT  3 strength tests  I./II. NR | Sign. improvement in TPC, PSQI, PASAT, and all 3 strength tests in favour of TG  AE: NR  FU: no* / 12 months  Contact by telephone: 68% continued the exercise program |
| Tomas-Carus et al.  2008 [67], 2009 [54] | ROB:  low  ITT: yes | T: 8 months  (3 days/week)  FU: no  University, Spain  outpatients | 17/16  women  age: 50.7/50.9  FMS 20.1 y  Pain 5.6 | 2/1 | TG: supervised exercise therapy in warm water (33°C), 1h incl. 10 min. warm up  CG: no treatment  CT: NR | Pain (Subscale FIQ)  FIQ  Physical fitness  SF-36  isokinetic strength  I./II. NR | Sign. improvement for pain, 7 of 8 subscales of SF36 and 5 of 7 fitness tests, 6 of 12 isokinetic strength tests, STAI, FIQ total in favour of TG at month 8  AE: NR |

| **TABLE 1: Hydrotherapy continued** | | | | | | | |
| --- | --- | --- | --- | --- | --- | --- | --- |
| Studies  Author, year | Risk of bias  ROB:  high/  unclear/  low  ITT  Intent- to-treat analysis  yes/no | Treatment  duration/  Follow-up  T: treatment  FU: follow-up  Setting, country  Inpatients/  outpatients | Sample  size  Treatment  group/  Control  group  Sex  Mean age  FMS: Duration/  years  Pain (VAS) | Dropouts  NR:  not  reported | Treatment  Treatment group (TG)  Control group (CG)  CT: Co-therapies, NM: not monitored | Outcome measures  FIQ: Fibromyalgia Impact Quest.  BDI: Becks Depr. Scale  VAS: Visual Analogue Scale  TPC: Tender Point Count  I. Primary outcome  II. Secondary outcome | Treatment efficacy and safety  AE: Adverse effects  NR: not reported |
| **Hydrogalvanic baths** | | | | | | | |
| Eksioglu et al.  2007 [57] | ROB:  low  ITT: yes | T: 8 weeks  (daily)  FU: 2 months  Hospital, Turkey, inpatients | 25/25  women  age: 45.1/39.1  FMS NR  Pain NR | 0/0 | TG: Hydrogalvanic bath, 20 min (37°C) daily for 10 sessions on a 2-week period and amitriptyline 10mg/d (8 weeks)  CG: Amitriptyline 10mg/d  CT: none | TPC  FIQ (QoL)  I./II. NR | TPC: no sign. difference between groups  FIQ: TG shows significant improvement in percent change of FIQ scores  AE: none |
| Günther et al.  1994 [61] | 2 | T: 5 weeks  (2 days/week)  FU: no  University, Austria  outpatients | 12/13  TG women  CG 10 w/3 m  age: 49.1/46.7  FMS 4 y  Pain 55.4  (0-100) | NR | TG: Hydrogalvanic bath, 20 min, followed by rest 30 min  CG: Jacobson relaxation  4 sessions/3 weeks with therapist  once a day/2 weeks without therapist/at home  CT: NR | Pain (VAS 0-100)  MPQ (German translation)  I./II. NR | No sign. differences between groups.  AE: NR |

Abbreviations used in Table 1: AIMS: Arthritis Impact Measurement Scales; ASES: Arthritis Self-Efficacy Scale; HADS: Hospital Anxiety and Depression Scale; HRQOL: health related quality of life; LTPAI: Leisure Time Physical Activity Instrument; MFI: Multidimensional Fatigue Inventory; MHI: Mental Health Inventory; MPI: Multidimensional Pain Inventory; MPI: Multidimensional Pain Inventory; MPQ: McGill Pain Questionnaire; PASAT: Paced Auditory Serial Addition Task; PGA/IGA: patient/investigator global assessment; PSQI: Pittsburgh’ Sleep Quality Index; QoL: Quality of Life; STAI: State-Trait Anxiety Inventory Questionnaire; SCI: Stress and Crisis Inventory; 6MWT: 6 Min Walk Test.
