# Supplementary material for: Therapeutic benefit of balneotherapy and hydrotherapy in the management of fibromyalgia syndrome: a qualitative systematic review and meta-analysis of randomized controlled trials
Source: Arthritis Res Ther. 2014 Jul 7;16(4):R141. doi: 10.1186/ar4603 (PMC4227103; doi:10.1186/ar4603)
Supplement: Additional file 2: Table S2 — Balneotherapy - characteristics of the included studies. Balneotherapy with the subgroups, mineral water (n = 3), spa therapy (n = 3), sulfur bath (n = 2), thalassotherapy (n = 1), phytothermotherapy (n = 1), mud (n = 1), acratothermal water (n = 1). Detailed study characteristics: author, year, risk of bias (high, unclear, low), intent-to-treat analysis (yes/no), sample size (treatment group/control group), sex, mean age, fibromyalgia syndrome (FMS) (duration/years), pain (visual analog scale, VAS), dropouts (n), treatment (treatment group/control group), co-therapies, outcome measures (primary/secondary outcome), treatment efficacy and safety (adverse effects). [file ar4603-S2.docx]

**TABLE 2: Balneotherapy - characteristics of the included studies.**

| Studies  Author, year | Risk of bias  ROB:  high/  unclear/  low  ITT  Intent- to-treat analysis  yes/no | Treatment  duration/  Follow-up  T: treatment  FU: follow-up  Setting, country  Inpatients/  outpatients | Sample  size  Treatment  group/  Control  group  Sex  Mean age  FMS: Duration/  years  Pain (VAS) | Dropouts  NR:  not  reported | Treatment  Treatment group (TG)  Control group (CG)  CT: Co-therapies, NM: not monitored | Outcome measures  FIQ: Fibromyalgia Impact Quest.  BDI: Becks Depr. Scale  VAS: Visual Analogue Scale  TPC: Tender Point Count  I. Primary outcome  II. Secondary outcome | | Treatment efficacy and safety  AE: Adverse effects  NR: not reported | | |
| --- | --- | --- | --- | --- | --- | --- | --- | --- | --- | --- |
|  |  |  |  |  | **Mineral/thermal water** | |  | |  |  |
| Ardiç et al.  2007 [69] | ROB: high  ITT: no | T: 3 weeks  (5 days/week)  FU: no  Spa resort, Turkey, outpatients | 12/12  women  age: 43.5/48.8  FMS 2 y  Pain 8.2 | 0/3 | TG: Thermal pool, 20 min (36°C)  CG: no treatment; waiting list  CT: Analgesics, non-steroidal antiinflammatory or antidepressive drugs were **not** permitted; NM | | Pain (VAS)  BDI  FIQ  TPC  PGE_2_ level  I./II. NR | | TG: significant improvements in all parameters at end of treatment (VAS, BDI, PGE2<0.001; TPC<0.01; FIQ<0.05)  AE: NR |  |
| Evcik et al.  2002 [72] | ROB:  high  ITT: yes | T: 3 weeks  (5 days/week)  FU: 6 months  University, Turkey, outpatients | 22/20  31 w/11 m  age: 42/41.5  FMS 1.3 y  Pain 7.2 | 0/0 | TG: Balneotherapy, 20 min (36°C)  CG: no treatment  CT: NSAID allowed; additional medication not allowed; NM | | Pain (VAS)  FIQ  TPC  BDI  I./II. NR | | TG: significant improvement in all parameters (p<0.001) between groups  FU: decrease in treatment effects, but still significant improvement in TPC (p<0.001), VAS and FIQ (p<0.005). BDI score similar to baseline (p>0.05)  AE: none |  |
| Yurtkuran and Celiktas  1996 [78] | ROB:  high  ITT: no | T: 2 weeks  (5 days/week)  FU: 2, 6 weeks  Rehabilitation Center, Turkey, outpatients | 20/20  37 w / 3m  age: 37.5/33.4  FMS NR  Pain 5.6 | NR | TG: Balneotherapy, 20 min (37°C), followed by relaxation exercises  CG: Relaxation exercises only  CT: No other medical or physical treatment; NM | | Pain VAS  Pressure algometer score  I./II. NR | | No between-group results  AE: none |  |

| **TABLE 2: Balneotherapy continued** | | | | | | | | | | |
| --- | --- | --- | --- | --- | --- | --- | --- | --- | --- | --- |
| Studies  Author, year | Risk of bias  ROB:  high/  unclear/  low  ITT  Intent- to-treat analysis  yes/no | Treatment  duration/  Follow-up  T: treatment  FU: follow-up  Setting, country  Inpatients/  outpatients | Sample  size  Treatment  group/  Control  group  Sex  Mean age  FMS: Duration/  years  Pain (VAS) | Dropouts  NR:  not  reported | Treatment  Treatment group (TG)  Control group (CG)  CT: Co-therapies, NM: not monitored | Outcome measures  FIQ: Fibromyalgia Impact Quest.  BDI: Becks Depr. Scale  VAS: Visual Analogue Scale  TPC: Tender Point Count  I. Primary outcome  II. Secondary outcome | | Treatment efficacy and safety  AE: Adverse effects  NR: not reported | | |
| **Sulphur bath** | | | | | | | | | |  |
| Buskila et al.  2001 [70]  Neumann et al.  2001 [53] | ROB: high  ITT: no | T: 10 days (daily)  FU: 1, 3 months  Rheumatology clinic  Location:  Dead sea; spa hotel, Israel, inpatients | 24/24  women  age: 54.6/54.3  FMS 11 y  Pain 8.0 | NR | TG: Balneotherapy, 20 min in sulphur pool (37°C, 2000mg/l sulphur)  CG: no treatment; staying at the same spa hotel with climate of the Dead Sea  CT: medication continued. No other co-therapies; NM | | Pain (VAS)  FIQ  TPC  Dolorimeter  FDI  SF-36  AIMS  HAQ (VAS)  I./II. NR | | TG: significant improvement for pain and TPC, persisting at 3 months (FU).  No sign. difference for other outcomes  AE: NR |  |
| Nugraha et al. 2011 [76] | ROB:  high  ITT: NR | T: 1 month  (3 days /week)  FU: 3 months  Univ. Hospital  Germany, outpatients | 19/13  women  age: 38.3/38.3  FMS NR  Pain NR | NR | TG: sulphur based bath (20.4 mg/l), head-out water immersion (36°C), 20 min  CG: tap water (same number and frequency of baths)  CT: medication continued .No co-therapies allowed; NM | | I. Lautenschläger pain score (LPS)  II. pressure (PPT),  II. heat (HPT),  II. cold pain thresholds (CPT) | | TG: sign. improvement for LPS and PPT after therapy between groups.  No sign. difference at FU and for HPT and CPT.  AE: NR |  |

|  |  |  |  |  | **TABLE 2: Balneotherapy continued** |  | |  | | |
| --- | --- | --- | --- | --- | --- | --- | --- | --- | --- | --- |
| Studies  Author, year | Risk of bias  ROB:  high/  unclear/  low  ITT  Intent- to-treat analysis  yes/no | Treatment  duration/  Follow-up  T: treatment  FU: follow-up  Setting, country  Inpatients/  outpatients | Sample  size  Treatment  group/  Control  group  Sex  Mean age  FMS: Duration/  years  Pain (VAS) | Dropouts  NR:  not  reported | Treatment  Treatment group (TG)  Control group (CG)  CT: Co-therapies, NM: not monitored | Outcome measures  FIQ: Fibromyalgia Impact Quest.  BDI: Becks Depr. Scale  VAS: Visual Analogue Scale  TPC: Tender Point Count  I. Primary outcome  II. Secondary outcome | | Treatment efficacy and safety  AE: Adverse effects  NR: not reported | | |
|  |  |  |  |  | **Spa therapy** | |  | |  |  |
| Dönmez et al.  2005 [71] | ROB:  unclear  ITT: no | T: 2 weeks  (6 days/week)  FU: 1,3,6,9 months  Recruitment Medical Faculty, Turkey  Location:  Spa centre, inpatients | 16/14  women  age: 43.3/43.1  FMS 11,5 y  Pain 6.75 | 0/1 | TG: Spa therapy, 20 min in thermal pool bath (mineral water 37°C);  followed by pressured shower (37°C) or classical massage every other day alternately, 15 min, stay at spa hotel  CG: no treatment; waiting list (continued to have their medical treatment and/or daily exercises)  CT: Regular medications were continued (all patients had plain analgesics, NSAIDs), no other co-therapies | | I. Pain (VAS)  I. FIQ  I. TPC  II. BDI  II. PGA | | TG: significant improvement in pain, TPC and FIQ after 2 weeks and 1 month and FIQ at 6 months.  AE: NR |  |
| Özkurt et al.  2011 [77] | ROB:  unclear  ITT: no | T: 2 weeks  (6 days/week;  2 daily baths)  FU: 1, 3 months  University, Turkey  Location:  Spa centre, inpatients | 25/25  women  age:  50.8/46.9  FMS 12.9 y  Pain 72 | 4/1 | TG: Balneotherapy, 2 x 20  min/day (thermomineral water, 36°C)  CG: no treatment; waiting list  CT: Education and exercise programmes continued.  Medical therapy continued, including analgesics, antidepressant drugs; NM | | FIQ BDI  PGA/IGA (VAS 0-100)  SF-36  Pain (VAS)  TPC  I./II. NR | | TG: sign. improvement for FIQ, BDI, pain, IGA, PGA, TPC except for 3 month IGA and 1 month BDI scores and for SF-36 vitality after therapy and at 3 months reported (but: statistical analysis published only for in between groups)  AE: NR |  |

| **TABLE 2: Balneotherapy continued – spa therapy** | | | | | | | | | | |
| --- | --- | --- | --- | --- | --- | --- | --- | --- | --- | --- |
| Studies  Author, year | Risk of bias  ROB:  high/  unclear/  low  ITT  Intent- to-treat analysis  yes/no | Treatment  duration/  Follow-up  T: treatment  FU: follow-up  Setting, country  Inpatients/  outpatients | Sample  size  Treatment  group/  Control  group  Sex  Mean age  FMS: Duration/  years  Pain (VAS) | Dropouts  NR:  not  reported | Treatment  Treatment group (TG)  Control group (CG)  CT: Co-therapies, NM: not monitored | Outcome measures  FIQ: Fibromyalgia Impact Quest.  BDI: Becks Depr. Scale  VAS: Visual Analogue Scale  TPC: Tender Point Count  I. Primary outcome  II. Secondary outcome | | Treatment efficacy and safety  AE: Adverse effects  NR: not reported | | |
| Zijlstra et al.  2005 [79] | ROB:  unclear  ITT: no | T: 15 days  (7-8 sessions of spa therapy)  FU: 3, 6, 12 months  Rheumatology clinics, Netherlands  Spa resort, Tunisia,  inpatients | 58/76  128 w/ 6 m  age: 48/47  FMS 10 y  Pain 5.9 | 0/1 | TG: Spa sessions (3 hours with Turkish bath, hot packs with algae, massage under a shower, whirlpool, underwater jetstream massage) and land and pool based exercise (7 x 1h), patient education (7 x 1h), recreational activities (luxurious touristic hotel)  CG: no treatment (treatment as usual, provided by their own physician in Netherlands)  CT: NR | | I. RAND-36 (SF-36)  II. FIQ, with pain VAS  II. MPQ  II. CIS (fatigue)  II. BDI  II. TPC  II. GTPS (graded tender point score)  II. 6MWT | | Sign. improvement for RAND 36 physical health subscale in favour of TG at FU after 3 months, but not at 6 and 12 months  FIQ, MPQ, CIS, GTPS sign better at 3 months and 6MWT at 12 months in favour of TG  No sign. difference between groups for other outcomes and other times  AE: mild sunburn, mild self limiting gastroenteritis, 1 sprained ankle visiting nearby village, 1 superficial injuries after falling off a horse |  |
|  |  |  |  |  |  | |  | |  |  |

| **TABLE 2: Balneotherapy continued** | | | | | | | | | | |
| --- | --- | --- | --- | --- | --- | --- | --- | --- | --- | --- |
| Studies  Author, year | Risk of bias  ROB:  high/  unclear/  low  ITT  Intent- to-treat analysis  yes/no | Treatment  duration/  Follow-up  T: treatment  FU: follow-up  Setting, country  Inpatients/  outpatients | Sample  size  Treatment  group/  Control  group  Sex  Mean age  FMS: Duration/  years  Pain (VAS) | Dropouts  NR:  not  reported | Treatment  Treatment group (TG)  Control group (CG)  CT: Co-therapies, NM: not monitored | Outcome measures  FIQ: Fibromyalgia Impact Quest.  BDI: Becks Depr. Scale  VAS: Visual Analogue Scale  TPC: Tender Point Count  I. Primary outcome  II. Secondary outcome | | Treatment efficacy and safety  AE: Adverse effects  NR: not reported | | |
|  |  |  |  |  | **Thalassotherapy** | |  | |  |  |
| de Andrade et al.  2008 [68] | ROB: Low  ITT: no | T: 3 months  (3 days/week)  FU: no  University, Brazil, outpatients | 23/23  women  age: 48.8/48.3  FMS NR  Pain 9.1 | 4/4 | TG: Thalassotherapy;  aerobic exercise in sea (temp. similar to pool, no waves), 60 min  CG: aerobic exercise in water pool (plain water 28-33°C), 60 min  Co-therapies: NR | | Pain (VAS)  Fatigue (VAS)  TPC  SF-36  PSQI (sleep)  BDI  I./II. NR | | **No differences between groups**, both improved significantly (p<0.05) in all parameters; only BDI showed a difference between groups (p<0.001) in favour of TG  AE: TG (11): muscle pain (8), sunburn (2), urinary infection (1)  CG (9): muscle pain (9) |  |
|  |  |  |  |  | **Mud** | |  | |  |  |
| Fioravanti et al.  2007 [74] | ROB:  high  ITT: yes | T: 2 weeks  (6 days/week)  FU: 4 months  Thermal spas, **multicenter study**, Italy, outpatients | 40/40  78 w/2 m  age: 46.2/48.6  FMS 2.2 y  Pain NR | 0/0 | TG: Mud-pack, 15 min (40-45°C) followed by immersion,10 min, in thermal water (37-38°C)  CG: no treatment (daily routines continued)  CT: Medication continued; only paracetamol additionally allowed; NM | | I.FIQ  I.TPC  I., minor symptoms of FMS VAS (0-100)  II. AIMS  II. HAQ | | TG: significant improvement in all parameters persisting at 4 months (FU), but no statistical intergroup analysis performed  AE: none |  |
|  |  |  |  |  |  | |  | |  |  |

|  |  |  |  |  | **TABLE 2: Balneotherapy continued** |  | |  | | |
| --- | --- | --- | --- | --- | --- | --- | --- | --- | --- | --- |
| Studies  Author, year | Risk of bias  ROB:  high/  unclear/  low  ITT  Intent- to-treat analysis  yes/no | Treatment  duration/  Follow-up  T: treatment  FU: follow-up  Setting, country  Inpatients/  outpatients | Sample  size  Treatment  group/  Control  group  Sex  Mean age  FMS: Duration/  years  Pain (VAS) | Dropouts  NR:  not  reported | Treatment  Treatment group (TG)  Control group (CG)  CT: Co-therapies, NM: not monitored | Outcome measures  FIQ: Fibromyalgia Impact Quest.  BDI: Becks Depr. Scale  VAS: Visual Analogue Scale  TPC: Tender Point Count  I. Primary outcome  II. Secondary outcome | | Treatment efficacy and safety  AE: Adverse effects  NR: not reported | | |
|  |  |  |  |  | **Phytothermotherapy** | |  | |  |  |
| Fioravanti et al.  2009 [73] | ROB:  high  ITT: yes | T: 11 days  (10 baths)  FU: 3, 6 months  University, Italy, thermal spa resort,  outpatients | 30/26  women  age: 53.2/48.6  FMS 2.2 y  Pain NR | 0/0 | TG: Phytothermotherapy; fermented hay (50-58°C) 20 min  CG: no treatment (daily routines continued)  CT: Medication continued; only paracetamol additionally allowed; NM | | FIQ  TPC  HAQ  AIMS (Italian)  I./II. NR | | TG: significant improvement in all parameters persisting at 6 months (FU), (p<0.001), but no statistical intergroup analysis performed  AE: none |  |
|  |  |  |  |  | **Acratothermal water** | |  | |  |  |
| Kesiktas et al.  2011 [75] | ROB:  high  ITT: yes | T: 3 weeks  (6 days/week)  FU: 6 months  Hospitals, Turkey, inpatients | 16/20*/20*  women  *matched to BT group (16) that was not randomized  age: 47/43/45  FMS 7.5 y  Pain7.2  FU  9/12/7 | 0/0/0  FU  7/8/13 | PTM: Physical Therapy Modalities,  15 sessions: TENS (15 min), Ultrasound (6 min), Infrared (15 min)  TG^1^: PTM + BT; 19 sessions, 20 min  (37-38°C), acratothermal water  TG^2^: PTM+ HT; 15 sessions, 20 min  (37°C), tap water  TG^3^: PTM  CT: Medication was discontinued throughout study period; at FU only paracetamol was permitted; NM | | Pain (VAS 0-10)  TPC  BDI  HDRS (depression)  dyspnea scale  spirometric measurements  QoL/VAS (0-10)  I./II. NR | | TG^1^ not random/TG^3^: sign. improvement in all parameters at the end of treatment and at FU.  TG^2^/TG^3^: Sign. improvement for pain, TPC, BDI, FEV1/FVC, HDRS after treatment and for pain, TPC, FEV1/FVC at 6 month FU.  AE: none |  |

Abbreviations used in Table: AIMS: Arthritis Impact Measurement Scales; CIS: Checklist Individual Strength; FDI: Functional Disability Index; HAQ: Health Assessment Questionnaire; HDRS: Hamilton Depression Rating Scale; HRQOL: health related quality of life; MPQ: McGill Pain Questionnaire; PGA/IGA: Patient/Investigator Global Assessment; PSQI: Pittsburgh’ Sleep Quality Index; QoL: Quality of Life; 6MWT: 6 Min Walk Test.
