# Supplementary material for: Therapeutic benefit of balneotherapy and hydrotherapy in the management of fibromyalgia syndrome: a qualitative systematic review and meta-analysis of randomized controlled trials
Source: Arthritis Res Ther. 2014 Jul 7;16(4):R141. doi: 10.1186/ar4603 (PMC4227103; doi:10.1186/ar4603)
Supplement: Additional file 3 — Risk of bias summary. The file contains authors’ judgements about each risk-of-bias item for each included study. Risk of bias: high, unclear, low. Items: selection bias (random sequence generation, allocation concealment, similar baseline characteristics); performance bias (blinding of participants and personnel); attrition bias (incomplete outcome data); reporting bias (selective reporting); detection bias (blinding of outcome assessment). BT, balneotherapy; HT, hydrotherapy; MW, mineral water; PBE, pool-based exercise; SB, sulfur bath; TT, thalassotherapy; Spa, spa center; Stanger, Stanger bath; Mud, mud bath; Hay, phytothermotherapy; PBE + E, pool-based exercise + education; PTM, physical therapy modalities (transcutaneous electrical nerve stimulation (TENS), ultrasound, infrared). [file ar4603-S3.docx]

**Additional file 3: Risk of bias summary.**
