# Supplementary material for: Therapeutic benefit of balneotherapy and hydrotherapy in the management of fibromyalgia syndrome: a qualitative systematic review and meta-analysis of randomized controlled trials
Source: Arthritis Res Ther. 2014 Jul 7;16(4):R141. doi: 10.1186/ar4603 (PMC4227103; doi:10.1186/ar4603)
Supplement: Additional file 4 — Risk of bias graph. The file contains authors’ judgement of each risk of bias item presented as percentages across all included studies. [file ar4603-S4.docx]

**Additional file 4: Risk of bias graph.**
