# Supplementary material for: Therapeutic benefit of balneotherapy and hydrotherapy in the management of fibromyalgia syndrome: a qualitative systematic review and meta-analysis of randomized controlled trials
Source: Arthritis Res Ther. 2014 Jul 7;16(4):R141. doi: 10.1186/ar4603 (PMC4227103; doi:10.1186/ar4603)
Supplement: Additional file 5 — Subgroup analysis for control group (LBE = land-based exercise; PBE = pool-based exercise; +E = education). The file contains the subgroup analysis regarding type of comparison group. [file ar4603-S5.docx]

**Additional file 5: Subgroup analysis for control group (LBE = land-based exercise; PBE = pool-based exercise; +E = education).**
