# Supplementary material for: Therapeutic benefit of balneotherapy and hydrotherapy in the management of fibromyalgia syndrome: a qualitative systematic review and meta-analysis of randomized controlled trials
Source: Arthritis Res Ther. 2014 Jul 7;16(4):R141. doi: 10.1186/ar4603 (PMC4227103; doi:10.1186/ar4603)
Supplement: Additional file 6 — Treatment effect of hydrotherapy (HT), balneotherapy (BT) and diverse therapies (hydrogalvanic bath (Stanger), mud therapy, sulfur bath (SB) and thalassotherapy (TT) on pain. The file contains the analysis of overall effects, taking into account all available studies, regardless of treatment modality. [file ar4603-S6.docx]

**Additional file 6: Treatment effect of hydrotherapy (HT), balneotherapy (BT) and diverse therapies (hydrogalvanic bath (Stanger), mud therapy, sulfur bath (SB) and thalassotherapy (TT) on pain.**
