# Supplementary material for: Therapeutic benefit of balneotherapy and hydrotherapy in the management of fibromyalgia syndrome: a qualitative systematic review and meta-analysis of randomized controlled trials
Source: Arthritis Res Ther. 2014 Jul 7;16(4):R141. doi: 10.1186/ar4603 (PMC4227103; doi:10.1186/ar4603)
Supplement: Additional file 7 — Sensitivity analysis for risk of bias (hydrotherapy (HT), pain). The file contains the forest plot displaying the relationship between effect size and risk of bias. [file ar4603-S7.docx]

**Additional file 7: Sensitivity analysis for risk of bias (hydrotherapy (HT), pain).**
