# Supplementary material for: Therapeutic benefit of balneotherapy and hydrotherapy in the management of fibromyalgia syndrome: a qualitative systematic review and meta-analysis of randomized controlled trials
Source: Arthritis Res Ther. 2014 Jul 7;16(4):R141. doi: 10.1186/ar4603 (PMC4227103; doi:10.1186/ar4603)
Supplement: Additional file 9 — Funnel plot (based on data of overall analysis, n = 17 studies). The file contains the scatter plot of the intervention effect estimates (SMD) from individual studies against their standard errors (SE). Publication bias may lead to asymmetry in funnel plots on visual inspection. [file ar4603-S9.docx]

**Additional file 9: Funnel plot (based on data of overall analysis, n = 17 studies).**
